# Diaqua­bis(2-methyl-1*H*-imidazol-3-ium-4,5-dicarboxyl­ato-κ^2^ *O*,*O*′)magnesium

**DOI:** 10.1107/S160053680902176X

**Published:** 2009-06-13

**Authors:** Yi Liang Li, Xin Guo, Ju Xian Wang, Yu Cheng Wang

**Affiliations:** a Institute of Medicinal Biotechnology, Chinese Academy of Medical Sciences and Peking Union Medical College, Beijing 100050, People’s Republic of China

## Abstract

The title compound, [Mg(C_6_H_5_N_2_O_4_)_2_(H_2_O)_2_], was prepared by reaction of Mg(NO_3_)_2_ and 2-methyl-1*H*-imidazole-4,5-dicarboxylic acid under hydro­thermal conditions. The Mg^II^ atom lies on an inversion centre and displays a distorted octa­hedral coordination geometry. An extended three-dimensional network of inter­molecular O—H⋯O and N—H⋯O hydrogen bonds stabilizes the crystal structure.

## Related literature

For the crystal structures of metal complexes with *N*-heterocyclic carboxylic acids, see: Nie *et al.* (2007 ▶); Liang *et al.* (2002 ▶); Net *et al.* (1989 ▶); Zeng *et al.* (2008 ▶).
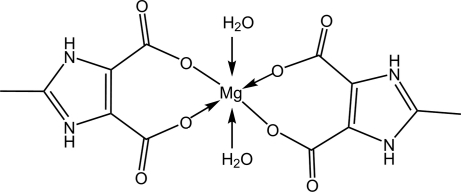


## Experimental

### 

#### Crystal data

[Mg(C_6_H_5_N_2_O_4_)_2_(H_2_O)_2_]
*M* *_r_* = 398.58
Triclinic, 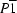

*a* = 4.943 (2) Å
*b* = 8.750 (6) Å
*c* = 9.621 (6) Å
α = 109.18 (3)°
β = 95.142 (17)°
γ = 93.14 (2)°
*V* = 389.9 (4) Å^3^
*Z* = 1
Mo *K*α radiation
μ = 0.18 mm^−1^
*T* = 292 K
0.30 × 0.25 × 0.20 mm

#### Data collection

Rigaku SCXmini diffractometer
Absorption correction: multi-scan (*CrystalClear*; Rigaku, 2005 ▶) *T*_min_ = 0.948, *T*_max_ = 0.967
4002 measured reflections
1767 independent reflections
1308 reflections with *I* > 2σ(*I*)
*R*_int_ = 0.046

#### Refinement

*R*[*F* ^2^ > 2σ(*F* ^2^)] = 0.061
*wR*(*F* ^2^) = 0.213
*S* = 1.19
1767 reflections
125 parameters
H-atom parameters constrained
Δρ_max_ = 0.39 e Å^−3^
Δρ_min_ = −0.42 e Å^−3^

### 

Data collection: *CrystalClear* (Rigaku, 2005 ▶); cell refinement: *CrystalClear*; data reduction: *CrystalClear*; program(s) used to solve structure: *SHELXS97* (Sheldrick, 2008 ▶); program(s) used to refine structure: *SHELXL97* (Sheldrick, 2008 ▶); molecular graphics: *SHELXTL*/*PC* (Sheldrick, 2008 ▶); software used to prepare material for publication: *SHELXTL*/*PC*.

## Supplementary Material

Crystal structure: contains datablocks I, global. DOI: 10.1107/S160053680902176X/rz2332sup1.cif

Structure factors: contains datablocks I. DOI: 10.1107/S160053680902176X/rz2332Isup2.hkl

Additional supplementary materials:  crystallographic information; 3D view; checkCIF report

## Figures and Tables

**Table 1:** Hydrogen-bond geometry (Å, °)

*D*—H⋯*A*	*D*—H	H⋯*A*	*D*⋯*A*	*D*—H⋯*A*
N1—H1⋯O4^i^	0.95	1.78	2.696 (4)	161
N2—H2⋯O2^ii^	0.95	1.81	2.727 (4)	162
O5—H5*B*⋯O2^iii^	0.93	2.23	3.155 (4)	172
O5—H5*B*⋯O1^iii^	0.93	2.36	2.961 (4)	122
O5—H5*A*⋯O3^iv^	0.87	1.98	2.841 (4)	170

Symmetry codes: (i) 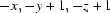; (ii) 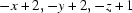; (iii) 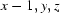; (iv) 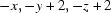.

**Fig. 1.**
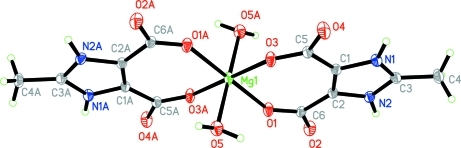
The molecular structure of the title compound, showing the atomic numbering scheme. Displacement ellipsoids are drawn at the 30% probability level. Atoms with suffix A are related to atoms with no suffix by 1-x, 2-y, 2-z.

**Fig. 2.**
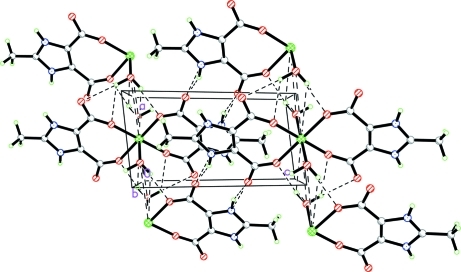
Packing diagram of the title compound viewed along the b axis. Intermolecular hydrogen bonds are shown as dashed lines.

## supplementary crystallographic information

### Comment

Recently, the study of metal complexes with *N*-heterocyclic carboxylic acids has been given considerable attention (Nie *et al.*, 2007; Liang *et al.*, 2002; Net *et al.*, 1989; Zeng *et al.*, 2008). In this paper, we report on the synthesis and crystal structure of the title compound, which was obtained by the hydrothermal reaction of Mg(NO_3_)_2_ with 2-methyl-1*H*-imidazole-4,5-dicarboxylic acid.

As shown in Fig. 1, the magnesium(II) atom, which lies on an inversion centre, adopts a distorted octahedral coordination, with the equatorial plane provided by four O atoms from two organic ligands [Mg1–O1 = 2.011 (2) Å; Mg1–O3 = 2.036 (2) Å] and the axial sites occupied by the O atoms of two water molecules [Mg1–O5 = 2.110 (3) Å]. The seven-membered chelate ring assumes an envelope-like conformation, with atom Mg1 displaced by 0.4353 (4) Å from the mean plane of the remaining atoms of the ring. The crystal structure is stabilized by intermolecular O—H···O and N—H···O hydrogen bonds (Table 1), forming an extended three-dimensional network (Fig. 2).

### Experimental

Colourless single crystals of title compound were obtained by hydrothermal treatment of Mg(NO_3_)_2_ (1 mmol), 2-methyl-1*H*-imidazole-4,5-dicarboxylic acid (1 mmol) and water (5 ml) over 4 days at 368 K. Yield: 67% (based on Mg(NO_3_)_2_.

### Refinement

The water H atoms and H atoms connected to N were located from a difference Fourier map but not refined [*U*_iso_(H)=1.5*U*_eq_(O, N)]. The methyl H atoms were placed at calculated positions and refined as riding, with C—H = 0.96 Å, and with *U*_iso_(H) = 1.5*U*_eq_(C).

### Crystal data

**Table:**

[Mg(C_6_H_5_N_2_O_4_)_2_(H_2_O)_2_]	*Z* = 1
*M**_r_* = 398.58	*F*(000) = 206
Triclinic, *P*1	*D*_x_ = 1.698 Mg m^−^^3^
Hall symbol: -P 1	Mo *K*α radiation, λ = 0.71073 Å
*a* = 4.943 (2) Å	Cell parameters from 1023 reflections
*b* = 8.750 (6) Å	θ = 3.9–27.4°
*c* = 9.621 (6) Å	µ = 0.18 mm^−^^1^
α = 109.18 (3)°	*T* = 292 K
β = 95.142 (17)°	Block, colourless
γ = 93.14 (2)°	0.30 × 0.25 × 0.20 mm
*V* = 389.9 (4) Å^3^	

### Data collection

**Table:**

Rigaku SCXmini diffractometer	1767 independent reflections
Radiation source: fine-focus sealed tube	1308 reflections with *I* > 2σ(*I*)
graphite	*R*_int_ = 0.046
Detector resolution: 13.6612 pixels mm^-1^	θ_max_ = 27.4°, θ_min_ = 2.7°
ω scans	*h* = −6→6
Absorption correction: multi-scan (*CrystalClear*; Rigaku, 2005)	*k* = −11→11
*T*_min_ = 0.948, *T*_max_ = 0.967	*l* = −12→12
4002 measured reflections	

### Refinement

**Table:**

Refinement on *F*^2^	Primary atom site location: structure-invariant direct methods
Least-squares matrix: full	Secondary atom site location: difference Fourier map
*R*[*F*^2^ > 2σ(*F*^2^)] = 0.061	Hydrogen site location: inferred from neighbouring sites
*wR*(*F*^2^) = 0.213	H-atom parameters constrained
*S* = 1.19	*w* = 1/[σ^2^(*F*_o_^2^) + (0.1078*P*)^2^ + 0.1465*P*] where *P* = (*F*_o_^2^ + 2*F*_c_^2^)/3
1767 reflections	(Δ/σ)_max_ < 0.001
125 parameters	Δρ_max_ = 0.39 e Å^−^^3^
0 restraints	Δρ_min_ = −0.42 e Å^−^^3^

### Special details

**Table:**

Geometry. All e.s.d.'s (except the e.s.d. in the dihedral angle between two l.s. planes) are estimated using the full covariance matrix. The cell e.s.d.'s are taken into account individually in the estimation of e.s.d.'s in distances, angles and torsion angles; correlations between e.s.d.'s in cell parameters are only used when they are defined by crystal symmetry. An approximate (isotropic) treatment of cell e.s.d.'s is used for estimating e.s.d.'s involving l.s. planes.
Refinement. Refinement of *F*^2^ against ALL reflections. The weighted *R*-factor *wR* and goodness of fit *S* are based on *F*^2^, conventional *R*-factors *R* are based on *F*, with *F* set to zero for negative *F*^2^. The threshold expression of *F*^2^ > σ(*F*^2^) is used only for calculating *R*-factors(gt) *etc*. and is not relevant to the choice of reflections for refinement. *R*-factors based on *F*^2^ are statistically about twice as large as those based on *F*, and *R*- factors based on ALL data will be even larger.

### Fractional atomic coordinates and isotropic or equivalent isotropic displacement parameters (Å^2^)

**Table:**

	*x*	*y*	*z*	*U*_iso_*/*U*_eq_	
Mg1	0.5000	1.0000	1.0000	0.0250 (4)	
C1	0.3793 (6)	0.7562 (4)	0.6015 (3)	0.0229 (7)	
C2	0.5912 (6)	0.8628 (4)	0.6010 (3)	0.0223 (7)	
C3	0.5089 (7)	0.6811 (4)	0.3730 (4)	0.0267 (7)	
C4	0.5167 (9)	0.5941 (5)	0.2137 (4)	0.0385 (9)	
H4A	0.4178	0.6492	0.1569	0.058*	
H4B	0.4347	0.4851	0.1885	0.058*	
H4C	0.7026	0.5915	0.1922	0.058*	
C5	0.1991 (7)	0.7356 (4)	0.7122 (3)	0.0240 (7)	
C6	0.7466 (6)	1.0094 (4)	0.7140 (3)	0.0233 (7)	
N1	0.3343 (6)	0.6454 (3)	0.4585 (3)	0.0268 (6)	
H1	0.2007	0.5554	0.4365	0.040*	
N2	0.6676 (6)	0.8120 (3)	0.4581 (3)	0.0258 (6)	
H2	0.7914	0.8719	0.4213	0.039*	
O1	0.6722 (5)	1.0584 (3)	0.8405 (2)	0.0319 (6)	
O2	0.9380 (5)	1.0749 (3)	0.6734 (3)	0.0380 (7)	
O3	0.2358 (5)	0.8310 (3)	0.8427 (2)	0.0312 (6)	
O4	0.0166 (6)	0.6225 (3)	0.6638 (3)	0.0410 (7)	
O5	0.2236 (5)	1.1757 (3)	1.0012 (3)	0.0336 (6)	
H5B	0.1354	1.1572	0.9070	0.050*	
H5A	0.0940	1.1836	1.0574	0.050*	

### Atomic displacement parameters (Å^2^)

**Table:**

	*U*^11^	*U*^22^	*U*^33^	*U*^12^	*U*^13^	*U*^23^
Mg1	0.0230 (8)	0.0287 (8)	0.0195 (8)	−0.0077 (6)	0.0050 (6)	0.0042 (6)
C1	0.0240 (15)	0.0222 (15)	0.0209 (15)	−0.0021 (12)	0.0033 (12)	0.0055 (12)
C2	0.0213 (14)	0.0243 (15)	0.0216 (15)	−0.0001 (12)	0.0050 (12)	0.0078 (12)
C3	0.0294 (17)	0.0240 (16)	0.0261 (16)	−0.0019 (13)	0.0057 (13)	0.0076 (13)
C4	0.054 (2)	0.0332 (19)	0.0227 (18)	−0.0042 (17)	0.0105 (16)	0.0014 (15)
C5	0.0232 (15)	0.0254 (15)	0.0224 (15)	−0.0035 (12)	0.0057 (12)	0.0067 (12)
C6	0.0230 (15)	0.0259 (16)	0.0206 (15)	−0.0033 (12)	0.0031 (12)	0.0081 (13)
N1	0.0277 (14)	0.0253 (14)	0.0248 (14)	−0.0064 (11)	0.0068 (11)	0.0052 (11)
N2	0.0284 (14)	0.0268 (14)	0.0228 (14)	−0.0031 (11)	0.0060 (11)	0.0092 (11)
O1	0.0353 (13)	0.0336 (14)	0.0227 (12)	−0.0103 (11)	0.0076 (10)	0.0049 (10)
O2	0.0346 (14)	0.0432 (16)	0.0330 (14)	−0.0165 (12)	0.0112 (11)	0.0098 (12)
O3	0.0319 (13)	0.0347 (14)	0.0208 (12)	−0.0114 (10)	0.0069 (10)	0.0022 (10)
O4	0.0408 (15)	0.0392 (15)	0.0323 (14)	−0.0239 (12)	0.0092 (12)	0.0008 (11)
O5	0.0280 (13)	0.0385 (14)	0.0326 (13)	−0.0015 (10)	0.0091 (10)	0.0090 (11)

### Geometric parameters (Å, °)

**Table:**

Mg1—O1^i^	2.010 (2)	C3—C4	1.475 (5)
Mg1—O1	2.010 (2)	C4—H4A	0.9600
Mg1—O3^i^	2.036 (2)	C4—H4B	0.9600
Mg1—O3	2.036 (2)	C4—H4C	0.9600
Mg1—O5^i^	2.110 (3)	C5—O4	1.238 (4)
Mg1—O5	2.110 (3)	C5—O3	1.249 (4)
C1—C2	1.365 (4)	C6—O2	1.237 (4)
C1—N1	1.388 (4)	C6—O1	1.247 (4)
C1—C5	1.496 (4)	N1—H1	0.9534
C2—N2	1.393 (4)	N2—H2	0.9489
C2—C6	1.498 (5)	O5—H5B	0.9297
C3—N2	1.330 (4)	O5—H5A	0.8656
C3—N1	1.336 (4)		
			
O1^i^—Mg1—O1	180.000 (1)	C3—C4—H4A	109.5
O1^i^—Mg1—O3^i^	89.92 (10)	C3—C4—H4B	109.5
O1—Mg1—O3^i^	90.08 (10)	H4A—C4—H4B	109.5
O1^i^—Mg1—O3	90.08 (10)	C3—C4—H4C	109.5
O1—Mg1—O3	89.92 (10)	H4A—C4—H4C	109.5
O3^i^—Mg1—O3	180.000 (1)	H4B—C4—H4C	109.5
O1^i^—Mg1—O5^i^	87.90 (11)	O4—C5—O3	124.6 (3)
O1—Mg1—O5^i^	92.10 (11)	O4—C5—C1	115.5 (3)
O3^i^—Mg1—O5^i^	88.95 (11)	O3—C5—C1	119.9 (3)
O3—Mg1—O5^i^	91.05 (11)	O2—C6—O1	124.7 (3)
O1^i^—Mg1—O5	92.10 (11)	O2—C6—C2	117.0 (3)
O1—Mg1—O5	87.90 (11)	O1—C6—C2	118.3 (3)
O3^i^—Mg1—O5	91.05 (11)	C3—N1—C1	110.7 (3)
O3—Mg1—O5	88.95 (11)	C3—N1—H1	129.8
O5^i^—Mg1—O5	180.000 (1)	C1—N1—H1	119.3
C2—C1—N1	105.8 (3)	C3—N2—C2	110.1 (3)
C2—C1—C5	136.3 (3)	C3—N2—H2	123.8
N1—C1—C5	117.9 (3)	C2—N2—H2	125.2
C1—C2—N2	106.6 (3)	C6—O1—Mg1	147.0 (2)
C1—C2—C6	135.2 (3)	C5—O3—Mg1	145.7 (2)
N2—C2—C6	118.2 (3)	Mg1—O5—H5B	111.2
N2—C3—N1	106.8 (3)	Mg1—O5—H5A	117.4
N2—C3—C4	127.0 (3)	H5B—O5—H5A	104.9
N1—C3—C4	126.2 (3)		
			
N1—C1—C2—N2	−0.3 (4)	N1—C3—N2—C2	−0.7 (4)
C5—C1—C2—N2	−179.6 (4)	C4—C3—N2—C2	177.5 (4)
N1—C1—C2—C6	−179.5 (3)	C1—C2—N2—C3	0.7 (4)
C5—C1—C2—C6	1.2 (7)	C6—C2—N2—C3	−180.0 (3)
C2—C1—C5—O4	176.8 (4)	O2—C6—O1—Mg1	−150.7 (3)
N1—C1—C5—O4	−2.4 (5)	C2—C6—O1—Mg1	30.6 (6)
C2—C1—C5—O3	−2.0 (6)	O3^i^—Mg1—O1—C6	142.4 (4)
N1—C1—C5—O3	178.8 (3)	O3—Mg1—O1—C6	−37.6 (4)
C1—C2—C6—O2	177.8 (4)	O5^i^—Mg1—O1—C6	53.4 (4)
N2—C2—C6—O2	−1.3 (5)	O5—Mg1—O1—C6	−126.6 (4)
C1—C2—C6—O1	−3.4 (6)	O4—C5—O3—Mg1	163.9 (3)
N2—C2—C6—O1	177.5 (3)	C1—C5—O3—Mg1	−17.4 (6)
N2—C3—N1—C1	0.5 (4)	O1^i^—Mg1—O3—C5	−151.8 (4)
C4—C3—N1—C1	−177.8 (3)	O1—Mg1—O3—C5	28.2 (4)
C2—C1—N1—C3	−0.1 (4)	O5^i^—Mg1—O3—C5	−63.9 (4)
C5—C1—N1—C3	179.3 (3)	O5—Mg1—O3—C5	116.1 (4)

Symmetry codes: (i) −*x*+1, −*y*+2, −*z*+2.

### Hydrogen-bond geometry (Å, °)

**Table:**

*D*—H···*A*	*D*—H	H···*A*	*D*···*A*	*D*—H···*A*
N1—H1···O4^ii^	0.95	1.78	2.696 (4)	161
N2—H2···O2^iii^	0.95	1.81	2.727 (4)	162
O5—H5B···O2^iv^	0.93	2.23	3.155 (4)	172
O5—H5B···O1^iv^	0.93	2.36	2.961 (4)	122
O5—H5A···O3^v^	0.87	1.98	2.841 (4)	170

Symmetry codes: (ii) −*x*, −*y*+1, −*z*+1; (iii) −*x*+2, −*y*+2, −*z*+1; (iv) *x*−1, *y*, *z*; (v) −*x*, −*y*+2, −*z*+2.
